# Mixed-species *Plasmodium falciparum* and *Plasmodium ovale* malaria in a paediatric returned traveller

**DOI:** 10.1186/1475-2875-13-78

**Published:** 2014-03-05

**Authors:** Heather Senn, Nadia Alattas, Andrea K Boggild, Shaun K Morris

**Affiliations:** 1 University of Toronto Faculty of Medicine, 1 King’s College Circle, Toronto, ON M5S 1A8, Canada; 2 Division of Infectious Diseases, Hospital for Sick Children, 555 University Ave., Toronto, ON M5G 1X8, Canada; 3 Tropical Disease Unit, Division of Infectious Diseases, University Health Network-Toronto General Hospital, 200 Elizabeth Street, Toronto, ON M5G 2C4, Canada; 4 Department of Medicine, University of Toronto, Suite RFE 3–805, 200 Elizabeth Street, Toronto, ON M5G 2C4, Canada; 5 Public Health Ontario Laboratories, 81 Resources Road, Etobicoke, ON M9P 3T1, Canada; 6 Department of Paediatrics, The Hospital for Sick Children, 555 University Avenue, Toronto, ON M5G 1X8, Canada

**Keywords:** Malaria, Traveller, *Plasmodium falciparum*, *Plasmodium ovale*, Mixed-species, Paediatric, Rapid diagnostic test

## Abstract

Malaria is a common and potentially fatal cause of febrile illness in returned travellers. Endemic areas for different malaria parasites overlap, but mixed species infections are rare. An adolescent male returned from a trip to Ghana in late summer 2013. He subsequently presented with blood smears positive for two species of malaria parasite, *Plasmodium falciparum* and *Plasmodium ovale,* on two isolated hospital visits within a six-week period. The epidemiology of mixed infections, likely pathophysiology of his presentation, and the implications for malaria testing and treatment in returned travellers are discussed.

## Background

Malaria remains the most commonly identified cause of fever in returned travellers, with the majority of infections acquired in sub-Saharan Africa [1]. Malaria can be caused by several different species of *Plasmodium* parasites with the largest burden of mortality and morbidity being due to *Plasmodium falciparum.* While *Plasmodium* species have different geographical distributions, there are areas of significant overlap. Thus, individuals may be infected with more than one species at any given time though this is rare in returned travellers [2]. Mixed infections, where more than one species are present either in the blood or liver stage of infection at one time, can have varied presentations. Different species have different clinical features and also require different treatments. Recently, a Canadian adolescent presented with symptoms of malaria twice within a six-week period to The Hospital for Sick Children (SickKids) in Toronto following a trip to West Africa. On both visits, he had multiple positive blood smears and each set was identified as a different *Plasmodium* species.

## Case presentation

A previously healthy 16-year-old male went on a volunteer trip to Ghana for three weeks in June and July, 2013. He did not take malaria prophylaxis. He had been on a similar volunteer trip to Kenya one year previously during which he took appropriate malaria prophylaxis and received hepatitis A, typhoid and yellow fever vaccinations prior to departure. Ten days after his return from Ghana, he presented to a local emergency department with a four-day history of fever, chills, headaches, nausea, and vomiting. A blood smear was positive for malaria parasites, later identified as *P. falciparum*, with a parasitaemia of 2.5% by the Public Health Ontario Laboratory (PHOL). *Plasmodium falciparum*-specific histidine rich protein-2 (HRP-2) was detected by rapid diagnostic test (RDT), while *Plasmodium* genus aldolase was undetectable. The patient was transferred to SickKids with findings of hypotension, elevated liver enzymes (Alanine aminotransferase (ALT) 134 U./L; aspertate aminotransferase (AST) 106 U/L; Gamma glutamyl transferase (GGT) 55 U/L), and thrombocytopaenia (platelets 19 × 10^9/L). A subsequent pre-treatment blood smear was positive for *P. falciparum* with 2.4% parasitaemia. He was treated with a three-day course of intravenous artesunate and oral atovaquone/proguanil. On day 5 of his admission he had clinically improved, and was discharged home with normalizing blood work trends and a negative blood smear. *P. falciparum* HRP-2 remained detectable by RDT despite an absence of parasitemia by thick and thin film microscopy. He was subsequently seen in follow-up in the Infectious Diseases clinic at SickKids one week later and reported to be asymptomatic with a repeat blood smear confirming an absence of parasitaemia from PHOL.

However, the patient returned to the emergency room at SickKids one month later with a two-day history of recurring symptoms of headache, nausea and vomiting without a fever. A blood smear was performed which was positive for malaria parasites with a parasitaemia level of <0.1%. The remainder of his laboratory results were within normal limits. It was initially thought that he was experiencing a recrudescence of *P. falciparum* malaria. Repeat blood smears were drawn on three consecutive days and were sent to PHOL for species identification while he was treated with a three-day course of oral atovaquone/proguanil. His symptoms were comparatively mild and resolved quickly and he was discharged home within 48 hours. He was followed up in clinic after four days, at which time species identification was available. All three blood smears identified *Plasmodium ovale* with <0.1% parasitaemia. Again, *P. falciparum* HRP2 was detectable by RDT, while *Plasmodium* genus aldolase was undetectable. Due to the discrepancy between microscopy and RDT, *Plasmodium* genus-specific and species-specific quantitative real time PCR (qPCR) targeting 18S rRNA was performed as described [3]. qPCR confirmed isolated *P. ovale* infection. The patient was treated with four doses of chloroquine and, after ruling out G6PD deficiency, he subsequently began a 14-day course of primaquine. Clinic follow-up two weeks after completing treatment confirmed he was doing well and a repeat blood smear at that time was negative.

## Discussion

Mixed species malaria infections are rare in travellers, comprising 2.1% of the 1,140 cases of malaria logged by the GeoSentinel Surveillance network based on treating physician diagnosis between 1997 and 2002 [2]. Mixed *P. falciparum* and *P. ovale* infections are especially uncommon, with only three instances (0.3%) reported in the above study. This statistic is in large part due to the rarity of *P. ovale* itself which accounted for only 5% of all malaria infections. A more recent study systematically tested over 1200 returned travellers from Italy who had at least 1 symptom of malaria with highly sensitive PCR testing. Of the 196 patients who tested positive for malaria, 3.1% had mixed-species infections including 2.0% testing positive for *P. falciparum* and *P. ovale. Plasmodium ovale* monoinfection accounted for 3.1% of malaria cases in this study [4]. In Ontario, over a six-year-period, 33 specimens from 29 patients with isolated *P. ovale* infection were identified by PHOL [3], underscoring the rarity of this infection in returned travellers. Mixed *P. ovale* and *P. falciparum* infections are also uncommon in areas where malaria is endemic. A study that followed 830 children in Burkina Faso from 2007–2010 found study participants had an overall malaria prevalence of 71% over the three years, and among those who presented with malaria, the rate of *P. falciparum* and *P. ovale* mixed infection was 0.72% [5]. *Plasmodium ovale* itself, again, was quite rare accounting for 1.08% of all recorded malaria infections.

While reported rates of *P. ovale* malaria are consistently low, there is evidence to suggest that infection with this species may actually be more common than most studies suggest. A research group in Senegal followed 101 children and 105 adults over a four-month malaria season with regular thick blood films regardless of the presence or absence of signs and symptoms of infection [6]. Although only three clinically significant *P. ovale* infections were documented, 48.5% of children and 32.5% of adults had blood films positive for *P. ovale* at some point during the study period suggesting that while infection with *P. ovale* may be common, the majority of infections are subclinical.

The two *Plasmodium* species have different life cycles and behaviours, accounting for the immediate and more severe presentation of *P. falciparum* malaria and delayed and milder course of *P. ovale* malaria in this patient. *Plasmodium falciparum* cycles through a transient liver phase, following which multiple schizonts rupture simultaneously and the infection proceeds to the symptomatic blood stage. This almost always occurs within two months of exposure. In contrast, *P. ovale* has a prominent liver hypnozoite phase. Parasites remain dormant in this stage and may persist for years. Presentation of *P. ovale* malaria is often delayed and hypnozoites rupture asynchronously producing intermittent symptoms of a milder nature. Specific treatment is required to target the hypnozoite phase to prevent relapse of infection. It is worth noting that the presentation of *P. ovale* and *P. falciparum* mixed infections cannot always be expected to follow this patient’s pattern. The timing of infection, variability of hypnozoite phase duration, interactions between species and other factors may play a role. A recent case report described a mixed-species infection where the patient presented first with *P. ovale* malaria with a subsequent delayed presentation of *P. falciparum*[7].

It is likely that the patient contracted both *Plasmodium* species during his recent trip to Ghana, with the presentation of *P. ovale* being either initially masked by the higher parasitaemia of *P. falciparum* or delayed while it remained in the hypnozoite phase. The possibility that this patient acquired his *P. ovale* infection during his trip to Kenya the year prior cannot be excluded. Although entomological data exists for co-infected mosquitoes, rates of confirmed mosquito co-infection are very low and most cases of mixed malaria infection are thought to be acquired from two separate inoculations [8]. Rapid diagnostic testing (RDT) is routinely performed as part of malaria screening to compliment blood smear identification in haematology and public health laboratories in Canada [9]. Although approved RDTs can detect *P. falciparum* and *Plasmodium vivax*, specific testing for *P. ovale* is not available. Current assays developed to detect mixed malaria infections have suboptimal sensitivity for the diagnosis of *P. ovale*[10,11]. An added disadvantage of RDTs is their prolonged detection of circulating antigen for weeks following effective malaria treatment [12], which can confuse the clinical picture. Persistent antigenemia, as seen in this case, can lead to an over-diagnosis of treatment failure, or misattribution of new symptoms to a relapsed or recrudesced prior infection.

The more sensitive malaria PCR, a costly test which is not routinely performed, is helpful in arbitrating incongruent results of microscopy and RDT, in identifying very low parasitemia infections, and in identifying those parasites with aberrant morphology on microscopy [4,13,14]. As described above, this test is available at PHOL, which uses one genus-specific and two duplex, species-specific real time quantitative PCR (qPCR) assays targeting the 18S rRNA region for the identification of *Plasmodium* species as described [3].

While appropriate prophylaxis would likely have prevented *P. falciparum* infection, it may not have prevented *P. ovale* infection. There have been case reports of returned travellers receiving appropriate prophylaxis who nevertheless present with *P. ovale* malaria [15,16]. This has also been reported for *P. vivax*, another *Plasmodium* species with a hypnozoite phase [17,18]. How frequently this occurs is unknown. Typical drugs used for prophylaxis such as atovaquone/proguanil do not successfully treat the liver phase of malaria infection, and any remaining liver schizonts can progress to the blood phase of infection once prophylaxis has stopped. The Centers for Disease Control thus recommend primaquine prophylaxis in areas of high *P. vivax* prevalence as well as a terminal prophylaxis in long-term travellers to endemic areas of relapsing malaria [19]. Relapses can occur up to several years after the initial infection for *P. ovale*, raising the possibility that while not likely, the patient described in this report may have contracted *P. ovale* during his previous trip to Kenya even though he took appropriate prophylaxis [20-23]. His recent symptoms could have been his first presentation, or he may have had previous reactivations that were not recognized as malaria. This highlights the importance of considering malaria in the differential diagnosis of symptomatic patients for months to years after travel to endemic areas, particularly if they present with a history of intermittent, recurrent illness.

In contrast to *P. falciparum*, *P. ovale* is almost universally chloroquine sensitive. Very few studies have attempted to determine the sensitivity of the blood stage of *P. ovale* to alternative therapies such as atovaquone/proguanil. One small study from 1996 treated seven adults presenting with *Plasmodium malariae* or *P. ovale* malaria with atovaquone and proguanil [24]. Each of the patients was cured of their infection. Another case report documented a single adult patient who cleared a *P. ovale* infection with atovaquone/proguanil [25]. The Centers for Disease Control guidelines for malaria treatment state that treatment regimens effective for *P. falciparum* are also acceptable for *P. ovale*[19]. Nevertheless, given the sparse data available, and because the patient’s most recent blood smear, taken on the last day of atovaquone/proguanil treatment, remained positive, the confirmed *P. ovale* infection was treated with chloroquine for 48 hours to clear the blood phase, followed by a two-week course of primaquine to clear the hypnozoite phase.

## Conclusion

In the summer of 2013, a paediatric returned traveller presented to SickKids with a rare, mixed-species malaria infection. He initially presented with more severe *P. falciparum* malaria, then returned one month later with milder symptoms and a blood smear positive for *P. ovale*. Mixed malaria infections are rare in returned travellers, and *P. ovale* itself is a rare cause of documented malaria that often has mild symptoms, delayed presentation, and may cause subclinical infections in the majority of cases. Compared to other species of malaria, little is known about this parasite’s actual prevalence and response to different anti-malarial therapies. This case highlights the value of accurate speciation of malaria blood films, and follow-up to ensure patients receive appropriate treatment including radical cure, if indicated, of the identified parasite. It also raises the possibility that more sensitive testing, such as PCR, may be useful in the setting of recurrent illness. It demonstrates the importance of considering malaria in the differential diagnosis of returned travellers with delayed symptoms, regardless of their use of prophylactic medications or previous malaria treatment. More generally, this case reinforces the importance of pre-travel physician counselling and adequate malaria prophylaxis for trips to endemic areas.

## Consent

Written informed consent was obtained from the patient for publication of this Case report. A copy of the written consent is available for review by the Editor-in-Chief of this journal.

## Competing interests

The authors declare that they have no competing interests.

## Authors’ contributions

HS drafted and revised the manuscript. NA and SM revised the manuscript and SM provided overall supervision. AKB critically appraised and revised the manuscript. All authors read and approved the final version.
